# Compliance towards infection prevention measures among health professionals in public hospitals, southeast Ethiopia: a cross-sectional study with implications of COVID-19 prevention

**DOI:** 10.1186/s41182-021-00318-y

**Published:** 2021-04-16

**Authors:** Demisu Zenbaba, Biniyam Sahiledengle, Abulie Takele, Yohannes Tekalegn, Ahmed Yassin, Birhanu Tura, Adem Abdulkadir, Edao Tesa, Alelign Tasew, Gemechu Ganfure, Genet Fikadu, Kenbon Seyoum, Mohammedawel Abduku, Tesfaye Assefa, Garoma Morka, Makida Kemal, Adisu Gemechu, Kebebe Bekele, Abdi Tessema, Safi Haji, Gebisa Haile, Alemu Girma, Mohammedaman Mama, Asfaw Negero, Eshetu Nigussie, Habtamu Gezahegn, Daniel Atlaw, Tadele Regasa, Heyder Usman, Adem Esmael

**Affiliations:** 1 Department of Public Health, School of Health Sciences, Madda Walabu University Goba Referral Hospital, Bale, Goba, Ethiopia; 2 Department of Midwifery, School of Health Science, Madda Walabu University Goba Referral Hospital, Bale, Goba, Ethiopia; 3 Department of Nursing, School of Health Science, Madda Walabu University Goba Referral Hospital, Bale, Goba, Ethiopia; 4 Department of Medicine and Surgery, School of Medicine, Madda Walabu University Goba Referral Hospital, Bale, Goba, Ethiopia; 5 Department of Anesthesia, School of Medicine, Madda Walabu University Goba Referral Hospital, Bale, Goba, Ethiopia; 6 Department of Medical Laboratory, School of Medicine, Madda Walabu University Goba Referral Hospital, Bale, Goba, Ethiopia; 7 Department of biomedical science, School of Medicine, Madda Walabu University Goba Referral Hospital, Bale, Goba, Ethiopia; 8 Department of Pharmacy, School of Medicine, Madda Walabu University Goba Referral Hospital, Bale, Goba, Ethiopia

**Keywords:** Compliance, COVID-19, Preventive measures, Southeast Ethiopia

## Abstract

**Introduction:**

The new coronavirus disease 2019 is an emerging respiratory disease caused by the highly contagious novel coronavirus which has currently overwhelmed the world. Realizing a comprehensive set of infection prevention measures is a key to minimize the spread of this virus and its impacts in all healthcare settings. Therefore, this study was aimed to assess the compliance towards COVID-19 preventive measures and associated factors among health professionals in selected public hospitals, southeast Ethiopia.

**Methods:**

A descriptive hospital-based cross-sectional study was conducted among 660 health professionals in public hospitals of southeast Ethiopia from October 1 to 31, 2020. A multistage sampling technique was used to select the study participants. Data were collected by interview using structured and pretested questionnaires. Ordinary logistic regression modeling was used to estimate the crude and adjusted odds ratio. To declare the statistical significance of factors associated with the outcome variable, *P*-value < 0.05 and 95% confidence interval were used.

**Results:**

A total of 654 health professionals were involved in the study; of whom, 313 participants were nurses. The overall good compliance and knowledge of health professionals regarding COVID-19 preventive measures were 21.6 and 25.5%, respectively. Working in the general hospital (AOR = 0.55; 95% CI 0.38, 0.79), service year (AOR = 2.10; 95% CI 1.35, 3.21), knowledge (AOR = 1.80; 95% CI 1.14, 2.89), and water availability (AOR = 3.26; 95% CI 2.25, 4.72) were some of the factors found to have a statistically significant association to compliance of health professionals regarding COVID-19 preventive measures.

**Conclusion:**

In this study, nearly one fifth of health professionals had good compliance towards COVID-19 prevention practices. Thus, a consistent supply of COVID-19 prevention materials, facilities, and improving the knowledge of health professionals through on and off-job training are crucial.

## Introduction

Viral diseases are persistent to emerge and denote a serious issue to public health. In the preceding centuries, numerous viral epidemics such as the severe acute respiratory syndrome coronavirus (SARS-CoV) and Middle East respiratory syndrome coronavirus (MERS-CoV) were identified in different periods [1]. In our time, the new coronavirus disease 2019 (COVID-19) caused by the highly contagious novel coronavirus (SARS-CoV 2) which has currently overwhelmed the world [2, 3]. The known modes of transmissions are droplets, contact, and aerosols [4].

The healthcare systems of the countries in the world became shaken and disturbed due to the COVID-19 pandemic [5]. In Africa, the COVID-19 pandemic is uncontrollably rising and the reactions to this pandemic have been challenged by a shortage of human resources, personal protective equipment (PPE), limited infrastructure, inadequate systems for the prevention, and fragile healthcare systems [6–8]. Globally, there have been more than nearly 80.3 million confirmed cases, and more than 1.8 million deaths registered up to December 26, 2020. In Ethiopia, the first patient with COVID-19 was detected on March 3, 2020, in Addis Ababa. From then until December 26, 2020, 121,880 COVID-19 confirmed cases and 1897 deaths were reported [9].

Health professionals (HPs) are at the front line in fighting coronavirus spread that put them at the risk of contracting COVID-19 [10, 11]. Keeping safety in the working environment for the health professionals and an operative plan is crucial in each phase of the pandemic. COVID-19 preventive measures recommended by World Health Organization (WHO) consist of regular handwashing, with soap and running water or using alcohol-based hand sanitizer; using face masks; avoid touching the eyes, nose, and mouth if hands are not clean; and avoiding close physical contact [12]. Realizing a comprehensive set of infection prevention measures in all healthcare settings is a collective responsibility and critical to protecting the health and lives of our precious healthcare workforce as well as the key to minimize the spread of the virus in both health care settings and the community [13].

Despite the greater destructive consequences of COVID-19 to individuals and public health, non-compliance to the preventive measures has been reported around the world [14–16]. Previous studies conducted in different countries indicated that around one-tenth of healthcare workers remove their masks while talking to the patient, four-fifths of them reused surgical masks, and 44.9% correctly dispose of the used facemask in the yellow-coded bags. Overcrowding, limited infection prevention materials/supplies, less commitment of healthcare providers to the policies and procedures, insufficient training, and lack of policy were factors affecting COVID-19 prevention practice [16–18].

In Ethiopia, multiple interventions were implemented to prevent the COVID-19 pandemic before it causes a substantial impairment to the community. The government declared a state of emergency, and established a COVID-19 taskforce at national who were informing disease prevention measures, deliver regular situational updates, and organize massive awareness creation efforts using diverse social and mass media stages. The COVID-19 catastrophe sends a solid message to resistant health systems that can only be realized with a committed health workforce. Protecting everyone requires urgently addressing shortages of health workers, updating infection prevention measures, investing in capacity building, and warranting safe working environments [19, 20].

Currently, there are no conclusive cure and specific antiviral therapeutics that are suggested for preventing or treating the COVID-19. Thus, preventive measures ranging from individuals to large-scale societal level practices are the only available means to control the spread of the virus and minimize its impacts [13, 21]. In this regard, the findings of this study are crucial to health professionals, health facilities, or higher administrators and researchers to halt the spread of COVID-19 [17, 22]. Therefore, this study was aimed to assess the compliance towards COVID-19 preventive measures and associated factors among health professionals in selected public hospitals, southeast Ethiopia, 2020.

## Methods

### Study settings and design

A descriptive hospital-based cross-sectional study was conducted in selected public hospitals found in three zones (Bale, East Bale, and West Arsi) of southeast Ethiopia from October 1 to 31, 2020. The purposively selected zones consist of 164 health centers and 12 hospitals (4 primaries, 6 general, and 2 referral public hospitals); of these, 7 hospitals were included in this study. According to Zonal Health Departments data of 2020, more than 4346 health professionals are employed with around 4.5 million populations in the catchment area of selected hospitals.

### Source and study population

All health professionals who were working in hospitals found in three zones (Bale, East Bale, and West Arsi) were the source population. And all health professionals who are working in selected hospitals of three zones were the study population.

### Sample size and sampling procedure

The sample size was calculated using the single population proportion formula by considering a 95% confidence interval (CI), 5% margin of error (*d*), proportion (*P*) 50%, and 1.5 of design effect. Considering 10% of the nonresponse rate, the final sample size 660 was obtained. A multistage sampling technique was used to select the study participants. Initially, the hospitals were stratified into primary, general, and referral hospitals. Accordingly, from each stratum, 2 primaries, 3 general, and 2 referral hospitals were selected using a simple random sampling technique. Based on the number of health professionals in each hospital, the total sample was proportionally allocated and each study participant was selected from the sampling frame using simple random sampling (lottery method).

### Data collection tool, technique, and quality control

The questionnaire was developed first in English and translated to local languages by an expert of both languages “Amharic” and “Afan Oromo” and then back to English to check for consistency of the translation. The questionnaire was adapted and modified in the local settings from formerly available studies [23–25], CDC [26, 27], and WHO guidelines [28].

Data were collected by interviewer-administered, structured, and pretested, questionnaires containing health professionals’ socio-demographic characteristics, compliance, and knowledge regarding COVID-19 preventive measures. To assure the data quality, data collection instruments were pretested on 5% of the total sample size, and to minimize over-reporting of compliance to COVID preventive measures, the questionnaire was also set in PK (practice and knowledge) order.

### Data processing and analysis

Data were entered using Epi-Data version 3.1 and analyzed using STATA version 14. Descriptive statistics were used to generate frequency tables, graphs, and ordinary logistic regression modeling was used to estimate crude (*p*-value < 0.25) and adjusted odds ratio. To declare the statistical significance of factors associated to outcome variables, *P*-values < 0.05 and 95% confidence interval were used.

### Operational definition

#### Compliance with COVID-19 preventive measures

In this study compliance to COVID-19 prevention measures was measured by using 18 questions with “Always, sometimes, and never” response options. A score of 1 was given for “always” and “0” for “sometimes and never” responses. Accordingly, the probable sum score of overall compliance to the COVID-19 prevention measure was ranged from 0 to 18. Respondents who score < 50% (score below 9), 50–79% (score 9–14 out of 18 items), and 80–100% (score 15–18 out of 18 items) were considered having poor, moderate, and good compliance towards COVID-19 prevention measures. Similarly, the knowledge of health professionals was assessed using 10 COVID prevention measure-related questions. Respondents who score < 50% (score below 5 out of total items), 50–79 %( score 5–7), and 80–100% (score 8–10) were considered having poor, moderate, and good knowledge towards COVID-19 prevention measures [29, 30].

*Adequate natural ventilation* is when working and staff room have at least two air inlets and outlet with room area 12 m^2^ and below.

*Functional handwashing facility* is functional handwashing sink with water and soap during data collection time.

## Results

### Socio-demographic characteristics of the study participants

Six hundred fifty-four health professionals were involved in the study with a 99% response rate, of which, 419 (64.1%) participants were males and 501 (76.6%) were in the age category of 25–35 years. Regarding health professionals, about 313 (47.9%) were nurses, and 318 (48.6%) were from the general hospital. About 227 (34.7%) and 264 (40.4%) of health professionals reported that working and staff rooms have no adequate natural ventilation. Regarding the COVID-19 prevention facility, 227 (34.7%) and 279 (42.7%) of health professionals reported that there were no functional handwashing facilities and continuous water supply at their workplace respectively (Table 1).

**Table 1:** Socio-demographic- and institution-related characteristics of health professionals in selected public hospitals, southeast Ethiopia (*n* = 654)

Variables	Frequency	Percent
**Levels of hospitals**		
Referral	293	44.8
General	318	48.6
Primary	43	6.6
**Working departments of respondents**		
Outpatient department	276	42.2
Inpatient department	288	44.0
Others	90	13.8
**Sex of respondents**		
Male	419	64.1
Female	235	35.9
**Age of respondents**		
≤ 24 years	74	11.3
25–35 years	501	76.6
36–45 years	62	9.5
≥ 46 years	17	2.6
**Profession of respondents**		
Doctors	87	13.3
Nurses	313	47.9
Midwives	98	15.0
Laboratory technicians (technologist)	37	5.7
Health officer	39	6.0
Others*	80	12.2
**Educational status of respondents**		
Diploma	137	20.9
First degree	490	74.9
Second degree and above	27	4.1
**Working rooms have adequate ventilation**		
Yes	427	65.3
No	227	34.7
**Staff rooms have adequate ventilation**		
Yes	390	59.6
No	264	40.4
**Presence COVID-19 prevention committee**		
Yes	474	72.5
No	166	25.4
I don’t know	14	2.1
**Functional handwashing facility**		
Yes	427	65.3
No	227	34.7
**Availability of continuous water supply**		
Yes	375	57.3
No	279	42.7
**COVID-19 training of respondents**		
Yes	269	41.1
No	385	58.9

Other working departments: clinics (dental, ART, psychiatric, ophthalmic, TB, and dermatology), X-ray, laboratory, operation room, pharmacy department, and triage

*Other health professionals: psychiatric nurse, BSc in optometry, radiographer, cataract surgeon, and dermatologist

### Compliance/practice to COVID-19 prevention measures

**Fig. 1:**
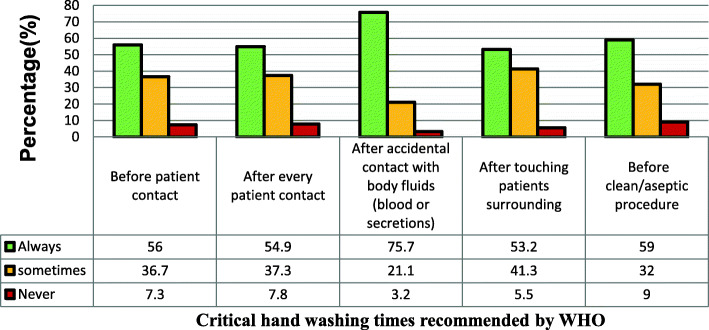
Handwashing practice/use sanitizer at WHO critical times among health professionals in selected hospitals of Southeast Ethiopia, 2020

**Fig. 2:**
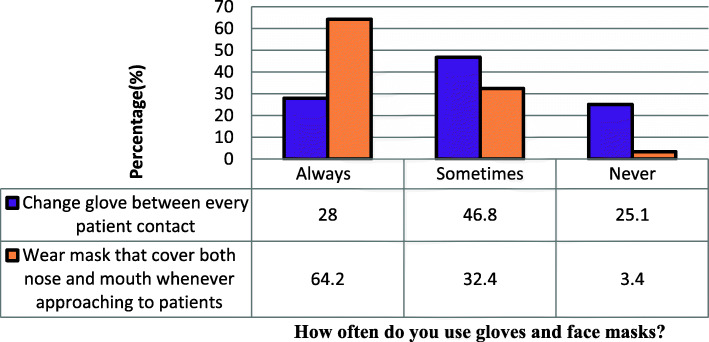
Glove and mask utilization among health professionals in selected public hospitals of Southeast Ethiopia, 2020

In this study, 141 (21.6%) of health professionals had good compliance with COVID-19 prevention measures. And the remaining 185 (28.3%) and 328 (50.2%) had moderate and poor compliance regarding COVID-19 preventive measures, respectively (Tables 2 and 3, Figs. 1 and 2).

**Table 2:** Compliance regarding COVID-19 preventive measure among health professionals in selected public hospitals, southeast Ethiopia, 2020 (*n* = 654)

Preventive measures	Level of compliance (how often)		
	Always, *n* (%)	Sometimes, *n* (%)	Never, *n* (%)
**Handwashing practice at WHO critical times**			
Wash hand/use sanitizer at arrival to workplace	347 (53.1)	287 (43.9)	20 (3.1)
Wash hand/use sanitizer before patient contact	366 (56.0)	240 (36.7)	48 (7.3)
Wash hand/use sanitizer after every patient contact	359 (54.9)	244 (37.3)	51 (7.8)
Wash hand immediately after accidental contact with body fluids (blood or secretions)	495 (75.7)	138 (21.1)	21 (3.2)
Wash hand/use sanitizer after touching patients surrounding	348 (53.2)	270 (41.3)	36 (5.5)
Wash hand with soap before clean/aseptic procedure	386 (59.0)	209 (32)	59 (9)
Wash hand/use sanitizer before leaving the workplace	324 (49.5)	279 (42.7)	51 (7.8)
**Handwashing with glove and mask utilization**			
Wash hand/use sanitizer before wearing sterile gloves	289 (44.2)	266 (40.7)	99 (15.1)
Wash hand/use sanitizer after removing gloves	297 (45.4)	290 (44.3)	67 (10.2)
Wash hand/use sanitizer before wearing a mask	253 (38.7)	304 (46.5)	97 (14.8)
Wash hand/use sanitizer before removing masks	251 (38.4)	297 (45.4)	106 (16.2)
Change glove between every patient contact	183 (28.0)	306 (46.8)	164 (25.1)
Wear a mask that covers both nose and mouth whenever approaching patients	420 (64.2)	212 (32.4)	22 (3.4)
**Disinfection practice and respiratory hygiene**			
Disinfect BP apparatus or thermometer after every patient contact	183 (28.0)	306 (46.8)	164 (25.1)
Disinfect examination bed or table between each service	225 (34.4)	296 (45.3)	133 (20.4)
Instruct symptomatic persons to cover mouth/nose when sneezing/coughing	407 (62.2)	223 (34.1)	24 (3.7)
Instruct symptomatic persons to dispose of used handkerchief during sneezing/coughing in a no-touch container	381 (58.3)	233 (35.6)	40 (6.2)
Open working room windows before starting regular activities	363 (55.5)	254 (38.8)	37 (5.7)

**Table 3:** The level of compliance to COVID-19 prevention measures among health professionals (HPs) in public hospitals, southeast Ethiopia, 2020 (*n* = 654)

Variables	HP’s compliance to COVID-19 preventive measures			***P***-value
	Poor, ***n*** (%)	Moderate, ***n*** (%)	Good, ***n*** (%)	
**Levels of hospitals**				
Referral	122 (41.6)	101 (34.5)	70 (23.9)	0.003
General	182 (57.2)	73 (23.0)	63 (19.8)	
Primary	24 (55.8)	11 (25.6)	8 (18.6)	
**Working departments of respondents**				
Outpatient department	134 (48.6)	79 (28.6)	63 (22.8)	0.07
Inpatient department	139 (48.3)	91 (31.6)	58 (20.1)	
Others	55 (61.1)	15 (16.7)	20 (22.2)	
**Sex of respondents**				
Male	220 (52.5)	115 (27.4)	84 (20.0)	0.25
Female	108 (46.0)	65 (27.7)	57 (24.3)	
**Age of respondents**				
≤ 24 years	46 (62.2)	18 (24.3)	10 (13.5)	0.02
25–35 years	253 (50.5)	142 (28.3)	106 (21.2)	
36–45 and above 46 years	29 (36.7)	25 (31.6)	25 (31.6)	
**Service years**				
≤ 2 years	134 (63.5)	56 (26.5)	21 (10)	< 0.001
3–6 years	99 (45.8)	63 (29.2)	54 (25.0)	
7–10 and above 10 years	95 (41.9)	66 (29.1)	66 (29.1)	
**Professions**				
Doctors and health officers	64 (50.8)	36 (28.6)	26 (20.6)	0.98
Nurses and midwives	208 (50.6)	115 (28.0)	88 (21.4)	
Lab technician and others	56 (47.9)	34 (29.1)	27 (23.1)	
**Educational status of respondents**				
Diploma	60 (43.8)	43 (31.4)	34 (24.8)	0.24
First degree and above	268 (51.8)	142 (27.5)	107 (20.7)	
**Presence COVID-19 prevention committee**				
Yes	199 (42.0)	148 (31.2)	127 (26.8)	< 0.001
No	129 (71.7)	37 (20.6)	14 (7.8)	
**Functional handwashing facility**				
Yes	160 (37.5)	137 (32.1)	130 (30.4)	< 0.001
No	168 (74.0)	48 (21.1)	11 (4.8)	
**Availability of continuous water supply**				
Yes	129 (34.4)	127 (33.9)	119 (31.7)	< 0.001
No	199 (71.3)	58 (20.8)	22 (7.9)	
**COVID-19 training**				
Yes	127 (47.2)	71 (26.4)	71 (26.4)	0.042
No	201 (52.2)	114 (29.6)	70 (18.2)	
**Knowledge of health professionals**				
Poor	82 (56.6)	40 (27.6)	23 (15.9)	0.015
Moderate	180 (52.6)	91 (26.6)	71 (20.8)	
Good	66 (39.5)	54 (32.3)	47 (28.1)	
**Overall level of compliance,** ***n*** **(%)**	**328 (50.2%)**	**185 (28.3%)**	**141 (21.6%)**

### Knowledge regarding COVID-19 preventive measures

In this study, 342 (52.3%) and 167 (25.5%) of health professionals had moderate and good knowledge regarding COVID-19 preventive measures respectively, whereas 145 (22.2%) of them had poor knowledge. The majority of the health professionals know respiratory droplets and close contact was the main transmission route of COVID-19. Concerning handwashing, only one fourth (25.9%) of health professionals correctly identify the recommended duration of handwashing by the World Health Organization (Table 4).

**Table 4:** Knowledge of health professionals regarding COVID-19 preventive measure in public hospitals, southeast Ethiopia, 2020 (*n* = 654)

Variable	Response	Frequency	Percent
COVID-19 is caused by	Viral	562	86.1
	Bacterial	67	10.3
	I don’t know	25	3.7
What is the main transmission route of COVID-19	Respiratory droplets and close contact	583	89.3
	Water	52	8.0
	Food	17	2.6
	I don’t know	2	0.2
How long is the incubation period for COVID-19	2–14 days	457	70.0
	3–7 days	92	14.1
	More than 14 days	101	15.5
	I don’t know	4	0.5
Who is susceptible to COVID-19	People are generally susceptible	404	61.9
	The old and children	97	14.9
	Young adults	69	10.6
	People with pre-existing disease	81	12.4
	I don’t know	3	0.3
The main clinical manifestations of COVID-19	Fever and dry cough	563	86.1
	Fatigue	42	6.4
	Sore throat and myalgia	43	6.6
	Diarrhea	6	0.8
Patient with underlying chronic disease are at a higher risk of infection	Yes	588	90.0
	No	44	6.7
	I don’t know	22	3.2
Treatment option for COVID-19	Supportive care	500	76.6
	Antiviral treatment provision	113	17.3
	No definitive management	37	5.7
	I don’t know	4	0.5
WHO recommended duration of handwashing with soap and water	40–60 s	169	25.9
	20–30 s	450	68.9
	1 h	35	5.2
WHO recommended duration of alcohol hand rub using sanitizer	*20–30 s*	229	35.1
	10 s	307	47.0
	20 s	118	17.9
Recommend physical distance	0–1 m	36	5.5
	2 and above m	618	94.5

### Factors associated with compliance towards COVID-19 prevention measures

Socio-demographic-, knowledge-, and health facility-related factors to compliance towards COVID-19 prevention measures were identified using an ordinary logistic regression model. Health professionals who working in general hospitals were 45% times less likely to have good compliance towards COVID-19 prevention measures than health professionals working in referral hospitals (AOR = 0.55; 95% CI 0.38, 0.79). The odds of having good compliance towards COVID-19 preventive measures were 2 times more likely among health professionals with 3–6 service years than health professionals who had ≤ 2 service years (AOR = 2.10; 95% CI 1.35, 3.21). Health professionals who have a good knowledge regarding COVID-19 preventive measures were 1.80 more likely to have good compliance regarding COVID-19 preventive measures than their counterparts (AOR = 1.80; 95% CI 1.14, 2.89). Similarly, the odds of having good compliance were nearly 3 times more likely among health professionals who know the presence COVID-19 Prevention Committee to their counterpart (AOR = 2.97; 95% CI 1.96, 4.50). The odds of having good compliance towards COVID-19 preventive measures were nearly 3 times higher among health professionals who had functional handwashing facilities (AOR = 2.66; 95% CI 1.78, 3.97) and continuous water supply (AOR = 3.26; 95% CI 2.25, 4.72) at their working place respectively (Table 5).

**Table 5:** Factors associated with compliance regarding COVID-19 prevention measures among health professionals in selected public hospitals, southeast Ethiopia (*n* = 654)

Variables/factors	Unadjusted and adjusted ordinary logistic regression				Base outcome (poor)
	COR 95% CI	P-value	AOR 95% CI	*P*-value	
**Level of hospital**					
Referral	1		1		
General	0.60 (0.45, 0.81)*	0.001	0.55 (0.38, 0.79)**	0.001	
Primary	0.62 (0.33, 1.14)	0.123	0.99 (0.49, 2.04)	0.99	
**Working departments**					
Outpatient department	1.48 (0.92, 2.39)	0.103			
Inpatient department	1.43 (0.89, 2.29)	0.14			
Others	1				
**Sex**					
Male	1				
Female	1.29 (0.96, 1.75)	0.09			
**Age**					
≤ 24 years	1				
25–35 years	1.63 (1.02, 2.65)*	0.049			
36–45 and above 46 years	2.84 (1.54, 5.25)*	0.001			
**Service years**					
≤ 2 years	1		1		
3–6 years	2.21 (1.52, 3.20)*	< 0.001	2.10 (1.35, 3.21)**	0.001	
7–10 and above 11 years	2.65 (1.84, 3.83)*	< 0.001	2.40 (1.50, 3.84)**	0.001	
**Educational status**					
Diploma	1				
First degree and above	0.75 (0.52, 1.06)	0.102			
**Presence COVID-19 prevention committee**					
Yes	3.61 (2.51, 5.19)*	< 0.001	2.97 (1.96, 4.50)**	< 0.001	
No	1		1		
**Functional handwashing facility**					
Yes	5.14 (3.63, 7.28)*	< 0.001	2.66 (1.78, 3.97)**	< 0.001	
No	1		1		
**Availability of continuous water supply**					
Yes	4.87 (3.52, 6.74)*	< 0.001	3.26 (2.25, 4.72)**	< 0.001	
No	1		1		
**COVID-19 training**					
Yes	1				
No	0.75 (0.56, 1.01)	0.056			
**Knowledge of health professionals**					
Poor	1		1		
Moderate	1.22 (0.84, 1.78)	0.29	1.17 (0.78, 1.78)	0.45	
Good	1.99 (1.30, 3.03)*	0.00	1.8 (1.14, 2.89)**	0.014	

*p < 0.05 = crude OR

**p < 0.05 = adjusted OR

## Discussion

Compliance with infection prevention practices is essential for minimizing transmission of infection, particularly crucial during the COVID-19 pandemic [19, 31]. This study has assessed the compliance towards COVID-19 preventive measures among health professionals in selected public hospitals, southeast Ethiopia. In this study, the overall good compliance towards COVID-19 preventive measures among health professionals was found to be 21.6%. This finding was lower than a study conducted in northwest Ethiopia, 38.7% [32], and in China, 87% [33]. The possible explanation for the disparity of this finding might be due to the difference in the number outcome variable category (two vs. three), distribution of COVID-19 prevention supplies and facilities (at different levels of hospitals, or poor managerial attention). Moreover, the compliance disparity might be due to the duration of the study (early and late pandemic) and disease burden.

In this study, about 64.2% of health professionals reported using face masks at the workplace whenever approaching COVID-19 suspected or confirmed patients. Despite wearing the face mask is mandatory within the health facility, our findings showed that only around two thirds of health professionals (HPs) use masks, which means, about one third of HPs significantly facilitate the chance to acquire or transmit the disease. This might be because some HPs may have difficulty of using masks due to uncomfortability and shortage of masks [29]. This finding is comparable with the study conducted in eight teaching hospitals in Ethiopia, 67.3% [34]. This consistency might be due to the shortage of masks which similarly affects all healthcare facilities found in Ethiopia. In this study, around 56% and 54.9% of health professionals’ practice frequent handwashing before and after patients contact respectively. Again, this finding is under expected; only half of HPs reported having compliance towards one of the critical handwashing times recommended by the World Health Organization. This might be due to 65.3 and 57.3% of health professionals reported having a functional handwashing facility and continuous water supply at their workplace, respectively. Regarding working environment disinfection, only 34.1 of HPs disinfect examination tables and beds found in working rooms before and after each procedure. This might be due to no access to disinfectants or less attention to the values of disinfection or believing as this activity belongs to cleaners or non-health professionals. This finding was slightly higher than the study finding, in Amhara Regional state Ethiopia, 29% [32]. This difference might be due to the inconsistency of health professional’s commitments regarding disinfection. Similarly, about 28% of HPs disinfect the blood pressure apparatus or thermometer after every patient contact. This might be due to the forgetfulness of health professionals or less attention, which may trigger the transmission of COVID-19 through direct contact.

This study shows that 25.5% of the health professionals have a good knowledge regarding COVID-19 prevention measures. This finding is lower than the study conducted, in Amhara Region, 70% [30]; northwest Ethiopia, 73.8% [32]; and in China, 88.4% [35]. The possible reason for this variation might be due to the study settings; participant’s negligence to focus on all items listed to evaluate knowledge, and only 41.1% of HPs taken training on COVID-19 prevention measures. This study indicated that the good compliance and knowledge of HPs regarding COVID-19 preventive measures were somewhat comparable, 21.6 and 25.5%, respectively. This implied that the health professional’s compliance towards COVID-19 preventive measures was not over-reported when compared to their knowledge.

Based on the ordinary logistic regression model; service years of health professionals, level of hospital, knowledge of health professionals, presence of the COVID-19 prevention committee, handwashing facility, and water availability were factors associated with compliance regarding COVID-19 preventive measures. The odds of having good compliance were nearly 1.8 times higher among health professionals who had good knowledge regarding COVID-19 preventive measures. This might be attributed to adequate training (duration of training), the presence of reading materials/internet services, and personal commitments. The odds of having good compliance towards COVID-19 preventive measures were 2 times more likely among health professionals with 3–6 service years than health professionals with ≤ 2 service years. This finding was comparable with the study finding in Amhara Region, Ethiopia [30]. This could be because as the HPs service years increase, the exposure and fear for such pandemic will increase and may practice prevention measures more strictly. On the other hand, the odds of having good compliance towards COVID-19 preventive measures were nearly 3 times more likely among health professionals who know the presence COVID-19 prevention committee in hospitals than their counterparts. This might be due to the committee informing HPs of the key COVID-19 preventive measures that are to be practiced during regular internal supervision.

### Limitation of the study

This study had some limitations that include hospitals currently serve as COVID-19 treatment and isolation centers were not included. As the study assessed self-reported compliance regarding COVID-19 prevention measures, it may be affected by social desirability. Despite these limitations, our findings provide valuable information about health professionals’ compliance regarding COVID-19 prevention measures.

## Conclusions

Regardless of the above limitation, around one fifth of health professionals in selected public hospitals of southeast Ethiopia had good compliance towards COVID-19 preventive measures. Nearly one fourth of health professionals had good knowledge of COVID-19 preventive measures. In this study, level of hospital, service years, presence of COVID-19 prevention committee, knowledge on COVID-19 preventive measures, availability of functional handwashing facility, and continuous water supply at the workplace were factors associated with compliance of health professionals towards COVID-19 preventive measures. Thus, a consistent supply of COVID-19 prevention materials, facilities, and improving knowledge of health professionals through on- and off-job training is recommended. Moreover, local health authorities should work in cooperation with fruitful stakeholders to fulfill gaps and monitor the operation of all COVID-19 preventive measures in hospitals.

## Data Availability

The datasets used and analyzed during the current study are available from the corresponding author on reasonable request.

## Abbreviations

COVID-19: Coronavirus disease 2019
HPs: Health professionals
WHO: World Health Organization
AOR: Adjusted odds ratio
COR: Crude odds ratio
